# An RFI-suppressed SMOS L-band multi-angular brightness temperature dataset spanning over a decade (since 2010)

**DOI:** 10.1038/s41597-023-02499-z

**Published:** 2023-09-08

**Authors:** Zhiqing Peng, Tianjie Zhao, Jiancheng Shi, Yann H. Kerr, Nemesio J. Rodríguez-Fernández, Panpan Yao, Tao Che

**Affiliations:** 1 State Key Laboratory of Remote Sensing Science, Aerospace Information Research Institute, Chinese Academy of Sciences, Beijing, 100101 China; 2 University of Chinese Academy of Sciences, Beijing, 100049 China; 3 National Space Science Center, Chinese Academy of Sciences, Beijing, 100190 China; 4 Centre d’Etudes Spatiales de la Biosphère (CESBIO), Université de Toulouse, Centre National d’Etudes Spatiales (CNES), Centre National de la Recherche Scientifique (CNRS), Institut de Recherche pour le Dévelopement (IRD), Institut national de recherche pour l’agriculture, l’alimentation et l’environnement (INRAé), Université Paul Sabatier, 18 av. Edouard Belin, bpi 2801, 31401 Toulouse, France; 5 Northwest Institute of Eco-Environment and Resources, Chinese Academy of Sciences, Lanzhou, 730000 China

**Keywords:** Hydrology, Hydrology

## Abstract

The Soil Moisture Ocean Salinity (SMOS) was the first mission providing L-band multi-angular brightness temperature (TB) at the global scale. However, radio frequency interferences (RFI) and aliasing effects degrade, when present SMOS TBs, and thus affect the retrieval of land parameters. To alleviate this, a refined SMOS multi-angular TB dataset was generated based on a two-step regression approach. This approach smooths the TBs and reconstructs data at the incidence angle with large TB uncertainties. Compared with Centre Aval de Traitement des Données SMOS (CATDS) TB product, this dataset shows a better relationship with the Soil Moisture Active Passive (SMAP) TB and enhanced correlation with *in-situ* measured soil moisture. This RFI-suppressed SMOS TB dataset, spanning more than a decade (since 2010), is expected to provide opportunities for better retrieval of land parameters and scientific applications.

## Background & Summary

The data from European Space Agency’s (ESA) and Centre National d’Etudes Spatiales (CNES) SMOS satellite are widely used in a variety of applications over both land and oceans. Applications over land include the water cycle (freeze/thaw^1^, soil moisture^2,3^, evapotranspiration^4^, precipitation^5^, net water flux^6^, etc.) and carbon cycle (proxy by vegetation optical depth, VOD)^7^, as well as applications for agriculture studies^8^. The SMOS satellite was launched on November 2, 2009, to a low-earth, polar sun-synchronous orbit with a mean altitude of 758-km, and flight forward with a fixed forward titled angle of 32.5° between the boresight and the local nadir. The SMOS satellite is equipped with a synthetical aperture radiometer, which is a Y-shaped structure with 69 antenna elements regularly distributed (0.875 wavelengths)^9^. This configuration ensures the unique capability of measuring multi-angular (about −10° to 60°) TB with 43-km average ground resolution over land (from 27-km at the nadir and limited to 55-km on the edges). Each pair of the small antennas measure the correlation of microwave radiation (at L-band, 1.4 GHz) for a given spatial frequency that depends on the relative position of the two antennas. With the correlations measured by all antenna pairs it is possible to form an image through a kind of inverse Fourier transform. The width of the resulting snapshot image at each integration step is about 1200 km, then the globe can be fully imaged twice every 3 days at 6:00 A.M. (ascending) and 6:00 P.M. (descending) local solar time at the equator.

To achieve the scientific objective of observing sea surface salinity over oceans and soil moisture over land with high accuracy, the SMOS satellite is well-calibrated^10^. Every elementary antenna of SMOS sees the sky above the Earth and the sky is aliased in the lower part of the images, since sky brightness temperature is relatively homogeneous and of known temperature, its impact can be corrected. The range of incidence angles changes as a function of the distance to the center of the swath, varying from 0° to 55° at the center to only angles in the 40° to 50° range at the swath’s extremities (see for instance Fig. 1 of Rodriguez-Fernandez *et al*.^11^). In addition, the image reconstruction noise and aliases on the edges induce noisier TB^10,12^, in particular for low incidence angles that are observed in the region of the field of view where the sky alias is corrected (the so-called “extended alias-free” region). Like other satellites, SMOS suffers from RFI contamination in certain areas of the world. Being the first L-band radiometer, problems were not expected in the protected band, so no filtering approaches were implemented in the receivers. This leads to data loss or meaningless retrievals of SMOS TBs^13,14^. Great success was obtained in reducing RFI problems since the SMOS launch, but many RFI sources still need to be located, filtered, or canceled, and new RFI sources also appear every day^15^. Therefore, apart from the flagging approaches developed at the Level 1 and 2 products in the SMOS processors, different kinds of methods have been proposed to post-process SMOS multi-angular TB. For example, a fixed width binned average has been used in the CATDS Level-3 (L3) daily SMOS multi-angular TB product, which also transforms the observed TB polarizations to a ground-based reference frame^16^. Some studies fitted multi-angular SMOS TBs with an exponential function^17^, a quadratic function^18^, or a third-order polynomial function^19^. Zhao *et al*. proposed a sophisticated method, named two-step regression, to refine the multi-angular SMOS TBs^20^. The approach was proposed based on a large number of simulations with a wide range of natural conditions using radiative transfer models. The two-step regression approach has been shown to perform best compared to other methods^21^ and has been used in the retrieval of soil moisture, snow density, and sea ice^22–25^.

**Fig. 1:**
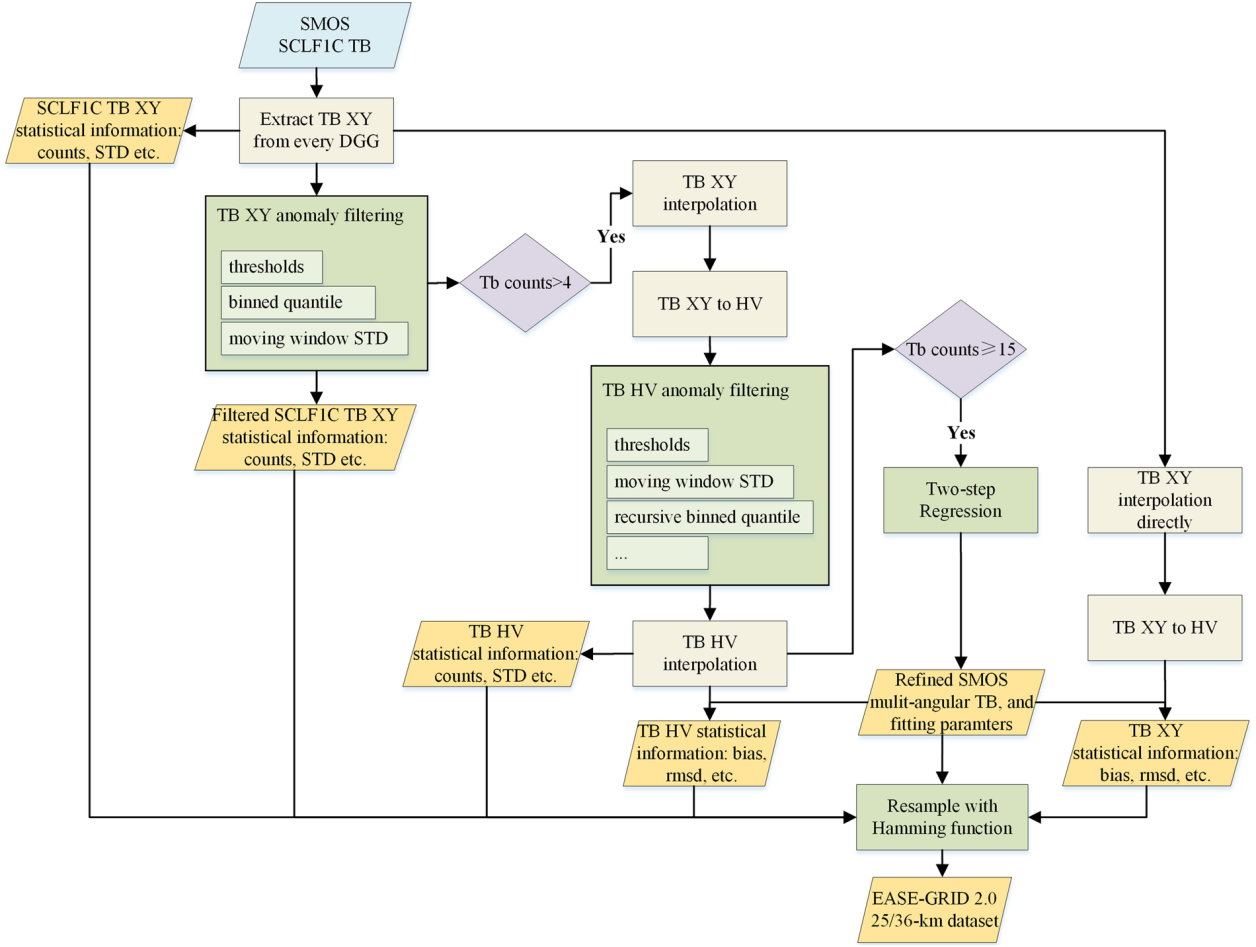
Flowchart of the two-step regression approach for SMOS multi-angular TBs refinement.

The current research aims to produce an RFI-suppressed SMOS multi-angular TB dataset using the two-step regression approach. This dataset is intended to provide improved opportunities for better scientific applications over land.

## Methods

The ESA SMOS Level-1C Full Polarization (SCLF1C) product^26^ from 2010 to 2021 (version 724) was refined using the two-step regression approach. The full polarized TB observations at the antenna frame are provided to 15-km in an equal-area discrete global grid (DGG) system (Icosahedral Snyder Equal Area projection, ISEA 4H9) in the SCLF1C product. The flowchart of the post-processor is shown in Fig. 1. It mainly contains four parts after extracting TBs at each DGG node from the SCLF1C orbit file.

Firstly, TB observations at the antenna frame were filtered with various criteria (details presented in antenna level rows in Table 1). The snapshot acquisition sequence in the full polarization mode is XX, XX/XY, YY, and YY/YX^27^. As there is no complete dataset for any epoch, a simple first-order spline interpolation was employed to fill the missing acquisitions. The anomaly filtering criteria comprise three steps: (1) TB thresholds filtering; (2) Binned quantile filter using 25% (*Q*_1_) and 75% quantile (*Q*_3_) with inter-quartile range (*IQR*), employing bins with a width of 5°, covering central angles ranging from 2.5° to 67.5°; (3) A time series anomaly detection using mean and standard deviation with a moving window size of 10 snapshots. Same as the latest CATDS L3 daily TB product (version 330)^28^, the RFI and the sun correction flag were not utilized in the procedure.

**Table 1:** The criteria used to filter TB before interpolation.

Reference frame	Filtering criteria	Details
TB XY (Antenna level)	Thresholds	$50K < T{B}_{X}\;and\;T{B}_{Y} < 340K$
		$$-50K < T{B}_{xy} < 50K$$
	Binned quantile	${Q}_{1}-1.5\cdot IQR < T{B}_{X}\;and\;T{B}_{Y} < {Q}_{3}+1.5\cdot IQR$
	Moving window standard deviation	$\overline{TB}-2\cdot STD < TB < \overline{TB}+2\cdot STD$
TB HV (Earth surface level)	Thresholds	$T{B}_{H}\le T{B}_{V}$
		$$50K < T{B}_{H}\;and\;T{B}_{V} < 340K$$
	Amplitude	$50K < \sqrt{T{B}_{H}^{2}+T{B}_{V}^{2}} < 500K$
	Moving window/Binned standard deviation	$\overline{TB}-2\cdot STD < TB < \overline{TB}+2\cdot STD$
	Binned quantile	${Q}_{1}-1.5\cdot IQR < T{B}_{H}\;and\;T{B}_{V} < {Q}_{3}+1.5\cdot IQR$

(Co-polarized *TB*_*X*_, *TB*_*Y*_ are antennal level TB, and *TB*_*xy*_ is the cross-polarized component; *IQR* is inter-quartile range determined by data in the fix bins or moving window, *Q*_1_ and *Q*_3_ are the corresponding 25% and 75% quantile respectively; $\overline{TB}$ and *STD* are the average and standard deviation of *TB* s at antennal level or earth surface level).

Then TBs at antenna level (X, Y) were converted to horizontal (H-) or vertical (V-) polarized TBs at the ground reference frame with the following equation:

1 $$\left[\begin{array}{c}T{B}_{H}\\ T{B}_{V}\\ T{B}_{3}\\ T{B}_{4}\end{array}\right]={\left[\begin{array}{cccc}{\cos }^{2}\left(\alpha \right) & {\sin }^{2}\left(\alpha \right) & \cos \alpha \cdot \sin \alpha  & 0\\ {\sin }^{2}\left(\alpha \right) & {\cos }^{2}\left(\alpha \right) & -\cos \alpha \cdot \sin \alpha  & 0\\ -\sin \left(2\alpha \right) & \sin \left(2\alpha \right) & \cos \left(2\alpha \right) & 0\\ 0 & 0 & 0 & 1\end{array}\right]}^{-1}\left[\begin{array}{c}T{B}_{X}\\ T{B}_{Y}\\ 2\cdot real(T{B}_{XY})\\ -2\cdot imag(T{B}_{XY})\end{array}\right]$$

where *α* = *θ*_*g*_ + *ω*_*f*_, *θ*_*g*_ is the geometric angle and *ω*_*f*_ is the Faraday rotation angle, *TB*_*H*_ and *TB*_*V*_ are H- and V-pol TBs, respectively; *TB*_3_ and *TB*_4_ are the Stokes 3 and Stokes 4 components, respectively.

Thirdly, a further filter procedure was applied to address potential outliers introduced during the interpolation procedure. For example, TB at H-polarization should be lower than TB at vertical polarization at non-nadir incidence theoretically^20^. The detailed filtering criteria at the ground reference are also listed in Table 1, with the same bin size as used at the antenna level filtering.

The final procedure involves the implementation of the two-step regression approach proposed by Zhao^20^. The first step used a quadratic function to obtain the TB at the nadir angle based on the finding from simulations that the total intensity of the electromagnetic wave is changed smoothly with the incidence angle. The quadratic function is shown in below:

2 $$T{B}_{H}+T{B}_{V}={\rm{A}}\cdot {{\rm{\theta }}}^{2}+{\rm{C}}$$

in which, θ is incidence angle, A and C are parameters fitted by non-linear Levenberg-Marquardt (LM) algorithm. C is the first Stokes parameter in the nadir angle, and half of C is used as a control point in the second step of the regression to avoid the polarization mixing, as it represents the TB value for both H- and V-polarization at the nadir angle. Another objective of Eq. (2) is to interpolate the TB trend affected by aliasing at small incidence angles.

The second step aims to obtain the multi-angular characteristics of H- and V-polarized TB using the following function separately:

3 $$\left\{\begin{array}{ccl}T{B}_{H} & = & {a}_{H}\cdot {{\rm{\theta }}}^{2}+\frac{C}{2}[{b}_{H}{\cdot \sin }^{2}({\rm{\theta }}){+\cos }^{2}({\rm{\theta }})]\\ T{B}_{V} & = & {a}_{V}\cdot {{\rm{\theta }}}^{2}+\frac{C}{2}[{b}_{V}{\cdot \sin }^{2}({d}_{V}\cdot {\rm{\theta }}){+\cos }^{2}({d}_{V}\cdot {\rm{\theta }})]\end{array}\right.$$

where C is adopted from Eq. (2); *a*, *b* and *d* are parameters fitted by LM algorithm, the subscript H and V represent H- and V-polarization respectively. Specific bounds were set for these parameters to ensure the desired trends of the curves. For example, parameter *b* denotes changes in the magnitude and direction of TB. It is set to be greater than 1 for V-polarization (*b*_*V*_) and less than 1 for H-polarization (*b*_*H*_) in the regression; *d*_*V*_ represents the Brewster angle effect in V-polarization with a lower bound of 1.

Following the above procedure, the refined TBs at any incidence angle ranging from 0° to 65° can be calculated with reduced RFI and aliasing effects. The interpolation allows for smooth estimation of refined TBs even at angles that were not directly observed in a snapshot.

To enable effective filtering and comprehensive evaluation of the two-step regression approach’s result and the impact of RFI according to different application requirements, various statistical metrics were computed at different steps, as illustrated in the yellow rhomboids in Fig. 1. This involved comparing the refined multi-angular TBs with two additional types of TBs:

(1) TBs used in applying the two-step regression approach.

(2) TBs directly interpolated at XY and converted to HV without any filtering using the procedure “TB XY interpolation directly” and the subsequent “TB XY to HV”.

For each comparison, both the overall and binned bias and root mean square deviation (RMSD) between the respective TBs and the fitted TB at corresponding incidence angles were calculated. The binned statistics were derived with a 5° width, covering central angles ranging from 2.5° to 62.5°, including an incidence angle of 40°.

For convenient application and better comparison with CATDS SMOS L3 daily TB and SMAP Level-2 TB in this study, the above-refined TBs and some auxiliary variables in the product files were resampled to a global Equal Area Scalable Earth Grid version 2 (EASE-GRID 2.0) 25-km and 36-km with a Hamming window approach using a footprint of 43-km.

To validate the refined multi-angular TB, they were compared with the TB from the CATDS L3 daily TB product^28^, generated from Level-1B snapshot products and projected to global EASE-GRID 2.0 25-km. The product provides fixed-angle binned TB with the above-mentioned fixed-width binned average method after lots of flag filtering and quality assessment^16^. The CATDS multi-angular products are in operation (OPER) and reprocess (RE07) modes. The RE07 mode product covers the period from 12 January 2010 to 24 May 2021, and the product in OPER mode ranges from 25 May 2021 till now.

SMAP is less affected by RFI because of RFI detection techniques applied to the hardware and software^29^. TB from SMAP L2 Radiometer Half-Orbit 36-km EASE-GRID Soil Moisture (SPL2SMP, version 8) with composite release ID of R18290^30,31^ was also used to compare with refined SMOS TB at 40°. SMAP TB products are projected to global EASE-GRID 2.0 36-km. To be noted, SPL2SMP uses the TB inherited from SMAP L1C Radiometer Half-Orbit 36-km EASE-GRID TBs (SPL1CTB, version 5). As the SCLF1C TB contains water bodies’ effect in the DGGs, the TB before adjustment for the presence of water bodies was extracted for comparison.

## Data Records

The refined SMOS multi-angular TBs dataset is located at National Tibetan Plateau Data Center (TPDC) and is provided in three types of projections^32^, including the ISEA4H9 grid and the EASE-GRID 2.0 25-km and 36-km grid system. All files are stored in the netCDF4 format. For the ISEA4H9 gridded data, it includes:

1. basic observation information, such as geolocations, DGG time, counts of strong or other types of RFI, and aliasing from the SCLF1C flag.
2. refined SMOS multi-angular TBs and corresponding flag range from 2.5° to 62.5°, with an interval of 5°, including TB and flag at the incidence angle of 40°.
3. abundant auxiliary statistics at the incidence angles of refined TB. These statistics comprise the overall and binned standard deviation of the original and filtered TBs, the bias, and the RMSD between the original, filtered TB, and the refined TB, as indicated in the yellow rhomboids in Fig. 1.
4. regression results, providing all parameters in Eqs. (2, 3), degree of freedom in the second step at H-polarization, and goodness of fit parameters such as the reduced chi-square^33^, Akaike and Bayesian information criterion^34^ statistics.

Additionally, the dataset in the EASE-GRID 2.0 projection contains variables that can be resampled to 25-km and 36-km grids, along with corresponding uncertainty. However, variables such as counts in (1) and regression results in (4) that cannot be resampled are not included in the dataset. To be noted, the resample method may introduce uncertainties in the results, and the standard deviation of a variable for resampling in an EASE-GRID 2.0 cell was used to evaluate the interpolation artifacts. More details can be found in the user guide.

The ISEA4H9 gridded filename was inherited from the SMOS SCLF1C orbit file with ascending or descending flag added in the filename. The EASE-GRID 2.0 grided files have a suffix about the resample method and grid size. All the datasets^32^ can be accessed at 10.11888/Terre.tpdc.300406 or https://cstr.cn/18406.11.Terre.tpdc.300406. Detailed description of the data records^32^ can be found in the readme.pdf file and codes and procedures for processing and plotting the figures are also included.

## Technical Validation

Below analyses are presented to support the technical quality of the refined SMOS multi-angular TB dataset.

### Demo of the two-step regression fitting procedure

**Fig. 2:**
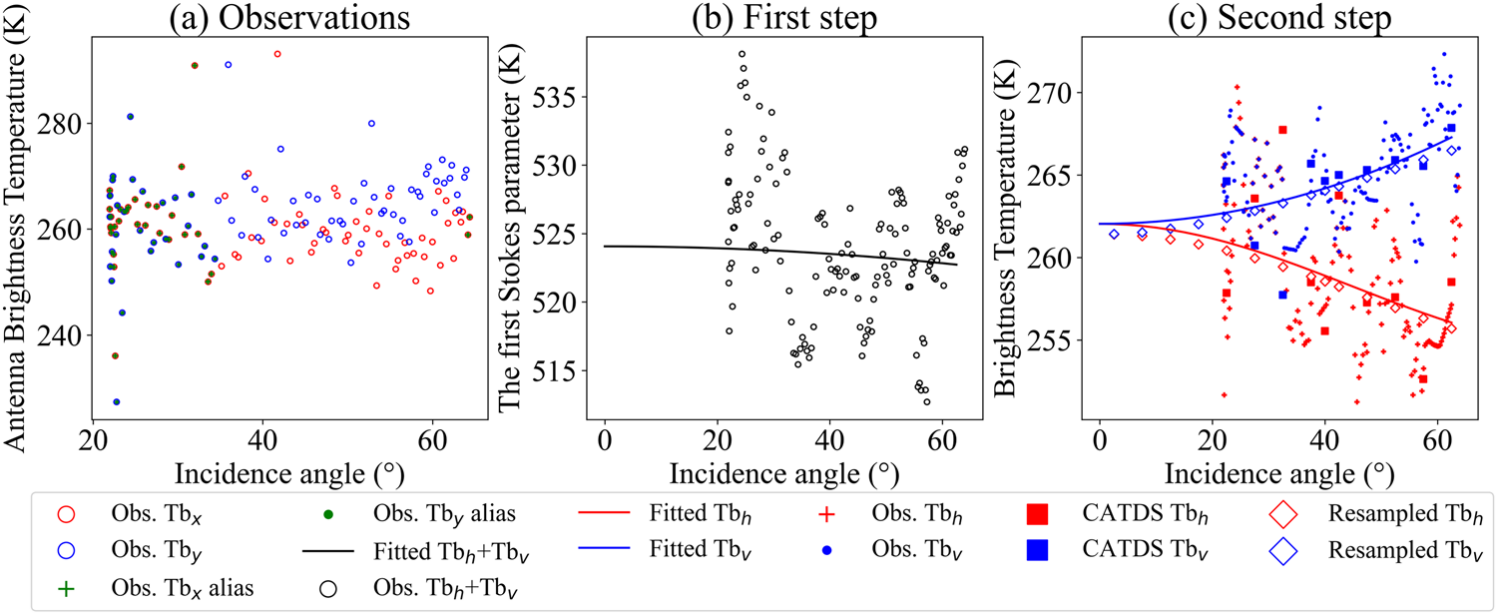
Fitting process of the two-step regression approach for SMOS multi-angular TBs of the DGG 4060112 on 2 June 2021. (**a**) The original observations at antenna level from SCLF1C; (**b**) The first step for obtaining TB at the nadir angle; (**c**) The second step for representing the multi-angular characteristics of the SMOS.

Figure 2 shows the fitting procedure of the two-step regression approach with the SCLF1C observations from the DGG id 4060112 (longitude: 104.591°E, latitude: 54.173° N) on June 2, 2021. Figure 2(a) shows aliasing exists in the snapshots with large oscillations, especially at small incidence angles in the “extended alias-free” field of view region. At this DGG node, aliasing at small incidence angles results in significantly higher total intensity for small incidence angles. However, the aliasing effect was effectively mitigated by the first-step regression procedure. This is primarily because the observations at intermediate and large incidence angles do not exhibit aliasing, which leads to its mitigation, as shown in Fig. 2(b). Figure 2(c) shows characteristics of the multi-angular TB and compares three results of multi-angular TB: the squares represent the CATDS OPER TB at EASE-GRID 2.0 25-km, two curves represent the two-step refined TB in ISEA4H9 DGG, and the rhombuses denote the resampled refined TB in EASE-GRID 2.0 25-km (same location with the CATDS TB). In theory, TBs at H-polarization decrease with incidence angles increase, and V-polarized TB would increase with incidence angles till the Brewster angle. Some abnormal condition exists for the CATDS OPER TB at 27.5° and 32.5° because V-polarized TBs are less than H-polarized TBs. This issue would cause data loss and other uncertainties for algorithms that need multi-angular TBs for soil moisture retrieval^23,24^. And CATDS TB exhibits more fluctuations than the refined TBs because CATDS TB was designed to keep characteristics of the real observations as much as possible, and the two-step regression was proposed to refine the TB for remote-sensed parameter retrieval. Despite these differences, all three types of TBs have the same multi-angular trend, which indirectly represents that the Hamming function resample method is reliable.

The multi-angular observation capability of SMOS also presents challenges for the two-step regression approach when observing areas with high heterogeneity (e.g., coastal regions; mountainous areas). In the land-ocean interface, there can be abrupt TB jumps between different incidence angles due to variations in surface properties. When low and intermediate incidence angles observe the land, large incidence angles predominantly capture ocean regions, leading to sudden decreases in TB. Similarly, in mountainous areas, the topographic effect can cause distinct changes in TB behaviour, e.g., the V-polarized TB tends to decrease, while the H-polarized TB increases^35^. These variations can impact the refined TB results. When TB jumps occur at several incidence angles, the two-step regression approach would smooth out these jumps or fail to achieve a robust refinement. If TB changes occur across the entire range of observations, the refined results may be shifted accordingly.

### Temporal variation of TBs at DGG samples

**Fig. 3:**
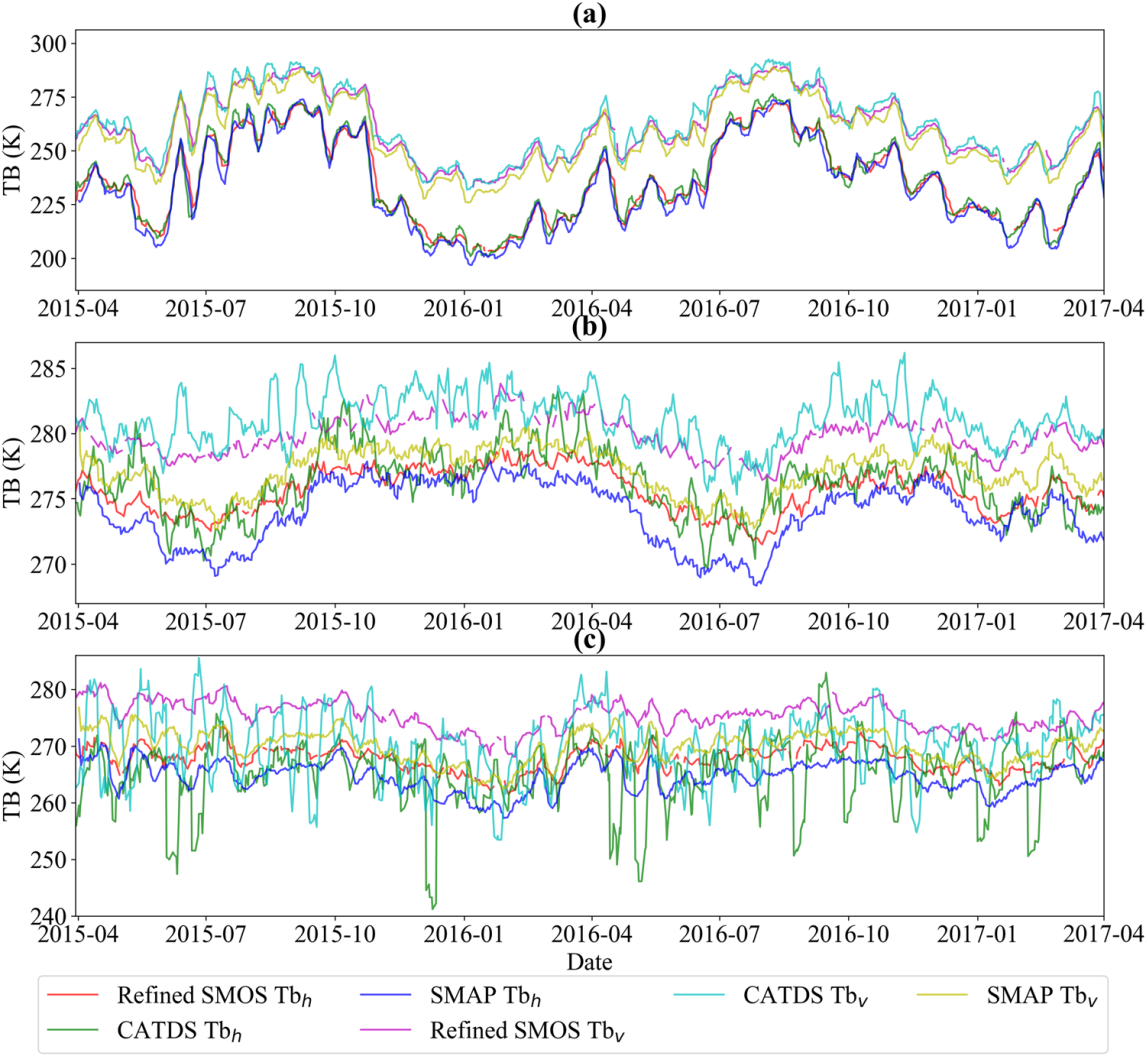
Time series of a 7-day moving average two-step refined SMOS TB, CATDS L3 TB and SMAP TB from 31 March 2015 to 1 April 2017. The title corresponding to the first column in Table 2.

Figure 3 illustrates the temporal variations of three TB products at an incidence angle of 40°: the two-step refined TB, the CATDS RE07 TB, and the SMAP TB in three selected grid points as listed in Table 2. To be noted, both the two-step refined SMOS TB and CATDS RE07 TB were resampled to EASE GRID 2.0 36-km with Hamming function. Figure 3(a) shows the time series of the two SMOS and SMAP TB at the Little Washita soil moisture network, all three types of TBs are consistent with each other, and the biases between them are very small, which indicates the performance of the two-step regression in situations of no RFI effect and the homogeneous low vegetated surface is reliable. Figure 3(b) is an example in Amazon Forest, where both SMOS products exhibit a warmer bias than SMAP TB for both H- and V-polarizations. Furthermore, the TB difference between SMOS polarizations (about 5.85 K) is greater than that of SMAP (about 3.21 K) most of time. This situation should be considered when analyzing subsequently retrieved parameters using the TBs from these two satellites. Figure 3(c) illustrates the performance of the two-step refined TB in a strong RFI-affected grid point. CATDS TBs at both polarizations exhibit greater fluctuations compared to the two-step regression refined TB, and the fluctuations are irregular, which may cause direct loss or less reliable results in parameter retrievals. It is worth noting that the refined TB may experience some data loss (as shown by discontinuities in Fig. 3) at certain times, which is caused by strong RFI (Fig. 3c) or small variations in multi-angular TBs due to dense vegetation (Fig. 3b), resulting in fitting failure or poor fitting quality.

**Table 2:** Information of the selected three grid points for TB comparison.

	Location	Longitude	Latitude	Climate zone	Dominate IGBP Class
(a)	North America	98.029° W	34.649° N	Temperate	Grasslands
(b)	South America	67.78° W	0.706° N	Tropical	Evergreen broadleaf forests
(c)	Asia	98.776° E	23.363° N	Temperate	Woody savannas

### Spatiotemporal coverage of TBs

**Fig. 4:**
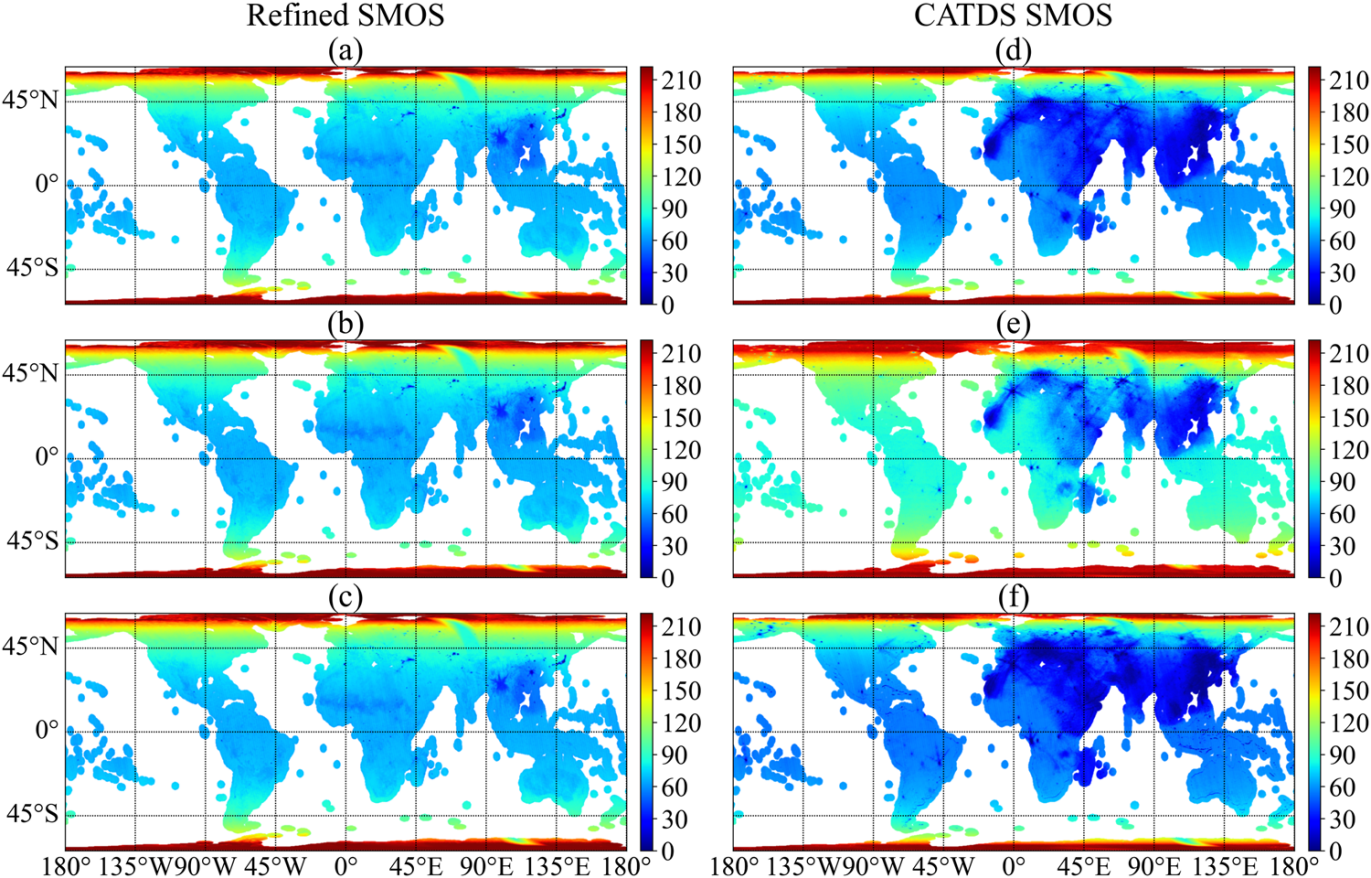
Spatial distribution of available count of H-polarized TB for refined SMOS (left column) and CATDS OPER SMOS (right column) in the ascending orbit. (**a**–**c**) refined SMOS TB counts at incidence angle of 27.5°, 40° and 52.5°; (**d**–**f**) CATDS OPER SMOS TB counts at incidence angle of 27.5°, 40° and 52.5°. The data period is from May 25 to December 31, 2021.

The spatiotemporal coverage of TBs is another important aspect of evaluating the RFI-suppression performance of refined TB. The available TB counts at incidence angles of 27.5°, 40° and 52.5° were calculated and presented in Fig. 4. It is worth noting that CATDS L3 TB provides daily TB data, whereas the refined TB is produced as an orbital product from the SCLF1C product. This may result in multiple overpasses of the same location and orbital direction within a single day, especially in regions with high latitudes. To ensure a fair and unbiased comparison of the spatiotemporal coverage between the two TB products, the available counts of refined TB at EASE GRID 2.0 25-km are consolidated to a single count (set as 1) when multiple overpasses occurred for the same location and orbital direction within a day. Regarding the RFI filtering, no RFI flags were used to produce CATDS L3 TB. Hence, a less strict RFI filtering criterion, as described in the CATDS quality assessment report^36^ was used to filter the CATDS L3 TBs. The filtering criterion is expressed as:

4 $$\frac{{\rm{Standard}}\_{\rm{Dev}}}{{\rm{Pixel}}\_{\rm{Radio}}\_{\rm{Accuracy}}} > 1.8$$

where Standard_Dev and Pixel_Radio_Accuracy are “Pixel_BT_Standard_Deviation” and “Pixel_Radiometric_Accuracy” in the CATDS L3 TB product, respectively. This less strict RFI filtering criterion was chosen because more strict filtering would lead to data loss (blank areas) as described in the report^36^. No RFI filtering in CATDS L3 TB would have greater available counts, but the unfiltered TBs may be less reliable, as indicated in Fig. 3(c).

Figure 4 demonstrates that CATDS L3 TB has more available counts at the intermediate incidence angle of 40° compared to lower and higher incidence angles. In regions with severe RFI, such as Asia, the available counts of refined TB are generally higher than those of CATDS. However, in other regions such as Australia and America, the available counts of CATDS TB are higher than those of the refined TB at intermediate angles. Nevertheless, the available counts of refined SMOS TB at difference incidence angles are very consistent, which is beneficial for remote-sensed algorithms that rely on multi-angular observations^23,24,37^.

### Validation of TBs at different incidence angles using *in situ* soil moisture at dense networks

Further, it is necessary to demonstrate the potential advantages of using this refined SMOS TB for soil moisture retrieval. The total emissivity was calculated using the refined SMOS TBs and compared with *in-situ* soil moisture from 19 dense networks (Table 3) at the same incidence angles as the CATDS L3 TB product, with the following equation:

5 $${e}_{P}^{\theta }=\frac{T{B}_{P}^{\theta }}{{T}_{s}}$$

where ${e}_{P}^{\theta }$ is the total emissivity at incidence angle *θ* and *P* polarization. *T*_*s*_ is the soil temperature extracted from the CATDS auxiliary data (https://data.catds.fr/cpdc/Common_products/GRIDDED/AUX/), projected in EASE GRID 2.0 25-km.

**Table 3:** The dense soil moisture networks information.

Network name	Country	Climate regime	Sensor numbers	Reference	Network name	Country	Climate regime	Sensor numbers	Reference
HOBE	Denmark	Temperate	24	^47,48^	Reynolds Creek	USA	Arid	18	^49^
iRON	USA	Continental	9	^50,51^	Fort Cobb	USA	Temperate	14	^52,53^
REMEDHUS	Spain	Temperate	20	^54^	Little Washita	USA	Temperate	18	^55^
Yanco	Australia	Semi-Arid	12	^56^	Little River	USA	Temperate	26	^57^
Kyeamba	Australia	Temperate	7	^58^	Shiquanhe	China	Cold	11	^59–61^
TERENO	Germany	Temperate	4	^62–64^	Maqu	China	Cold	9	
Walnut Gulch	USA	Arid	29	^65^	Naqu	China	Polar	30	^66,67^
St. Joseph’s	USA	Cold	10	^68^	Pali	China	Tundra	9	
South Fork	USA	Cold	20	^69^	FMI	Finland	Code	14	^70^
BIEBRZA-S-1	Poland	Continental	17	^71,72^					

Sensor numbers refers to the maximum station of a network in the validation period, if two stations are installed at the same location (same longitude and latitude, but different sensors), it is treated as one.

**Fig. 5:**
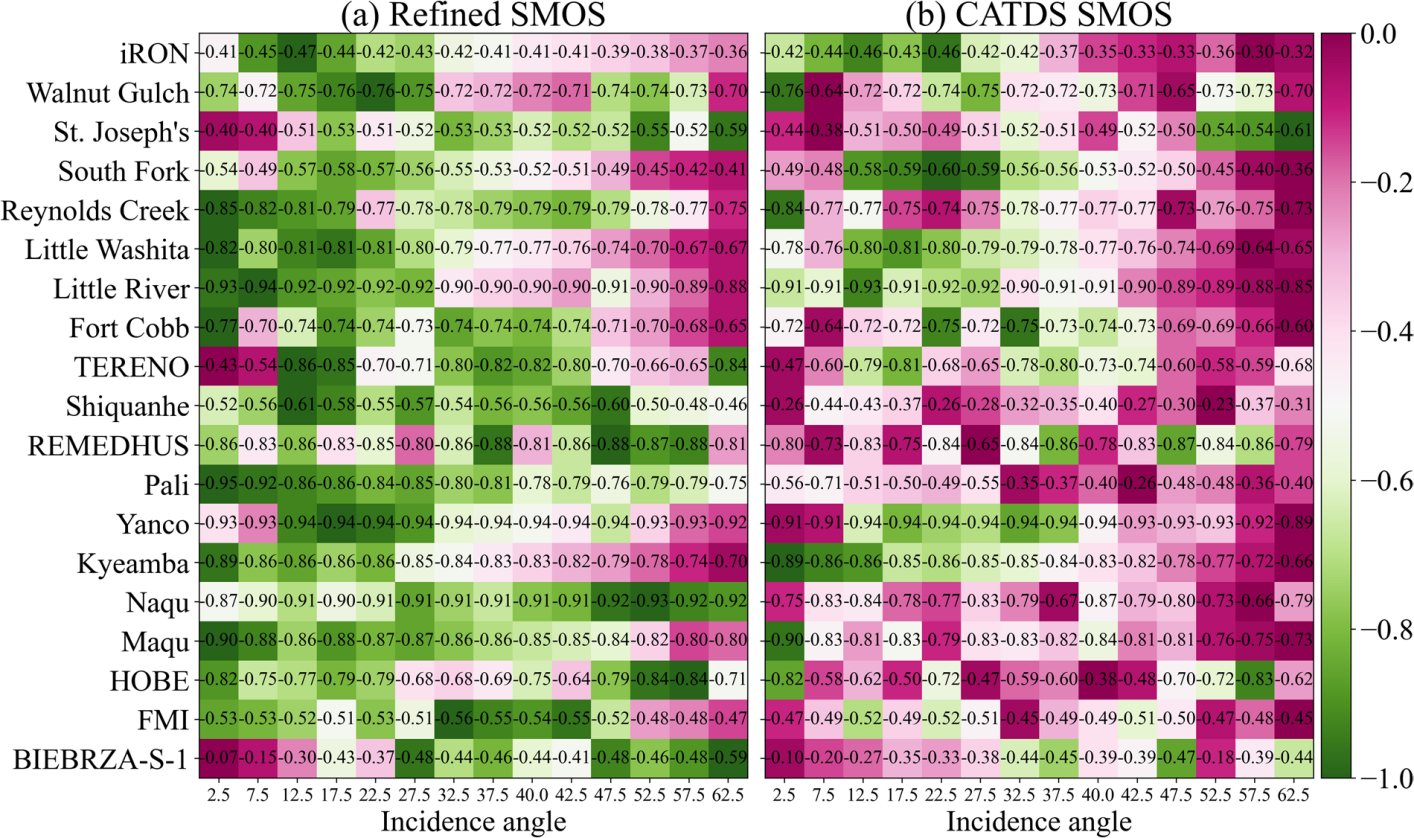
R between emissivity (H-polarization) and soil moisture of multi-angular refined and CATDS SMOS in comparison with *in-situ* soil moisture networks in 2016. The colour is scaled row-wise to values from 0 to 1. For every row, the better linearity is represented in green. For instance, to illustrate the interval of change in R ranging from −0.73 to −0.85 in the Reynolds Creek network, it was stretched to a range from 0 to 1, and the value −0.73 is represented in purple, and the value −0.85 is depicted in green.

For each soil moisture observation network, a bounding rectangle was defined using the maximum and minimum longitude and latitude of network stations. The emissivity of the EASE-GRID 2.0 cells located within this rectangle was averaged and compared with the average soil moisture for validation. To be noted, the Pearson’s correlation coefficients (R) were calculated based on the same available counts, and a weak RFI filtering criterion, as described in Eq. (4), was used to filter CATDS L3 TBs. The R values between H-polarized emissivity and soil moisture were shown in Fig. 5, and the V-polarized emissivity has a similar performance (not shown here). The results indicate that the two-step regression refined SMOS TB may outperform CATDS RE07 TB in most cases for soil moisture retrieval because the R values (negative) between emissivity and soil moisture of refined TB are stronger compared to CATDS L3 TB, indicating a better relationship between emissivity and soil moisture^38^. Additionally, the application of the multi-channel collaborative algorithm (MCCA)^39^ to multi-frequency observations, such as those from the Advanced Microwave Scanning Radiometer (AMSR)^40^, has revealed a notable trend in VOD: VOD values increase with frequency for each land cover type. This finding provides valuable insights into the characteristics of VOD across different frequencies and land cover conditions. This dataset may be promising to analyse the polarized characteristics of VOD using mono-polarization-multi-angular observations based on the MCCA.

### Spatial comparison of the refined TB at 40° with SMAP TB

**Fig. 6:**
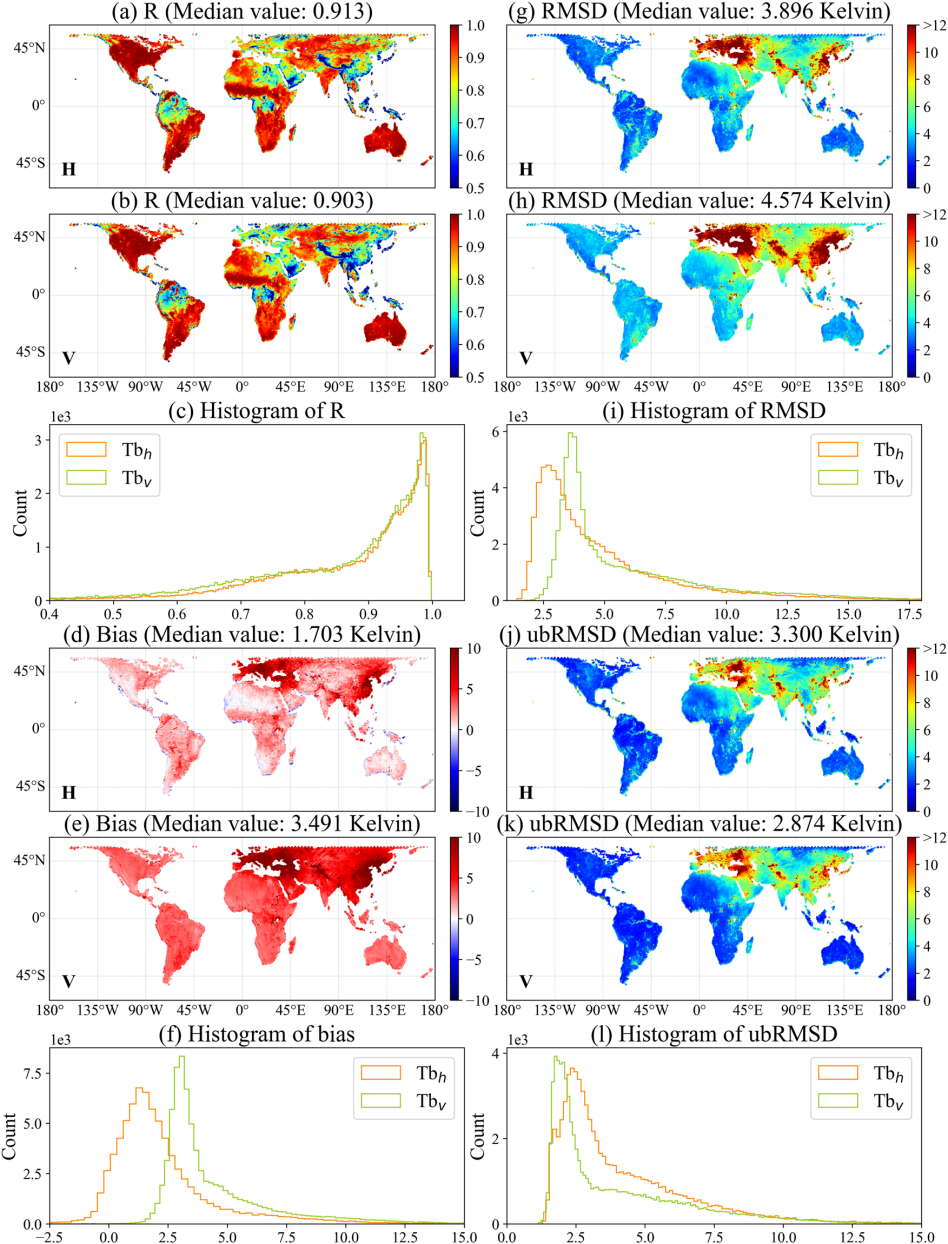
Grid-based statistics of R (**a**–**c**), Bias (**d**–**f**), RMSD (**g**–**i**), and ubRMSD (**j**–**l**) between the two-step regression approach refined SMOS TB (resampled to EASE-GRID 2.0 36-km) at 40° and SMAP TB. The data period is from 31 March 2015 to 31 December 2021.

To illustrate the performance of the refined SMOS TB dataset, TB at 40° (resampled to EASE-GRID 2.0 36-km) was compared with the SMAP TB product. To assess the correlation and differences between the two datasets, R, bias (unit: K), RMSD (unit: K), and unbiased root mean square deviation (ubRMSD, unit: K)^41^ were used as evaluation metrics. Figure 6 shows the details of the above statistics. The comparison spans from 31 March 2015 to 31 December 2021. While SMOS and SMAP cross the equator at the same time, they pass over an area at higher latitudes either before or after 6 am/pm. This results in overlapping orbits at a certain time. To ensure a fair comparison, a threshold of 3600 seconds was used to filter the overlap area. Additionally, SMAP flags including “Static Water”, “Radar-derived Water Fraction”, “Coastal Proximity” and “Urban Area” were used to filter the TB. The results show that most regions of the globe have strong correlation values (R > 0.9) between the refined SMOS TB and SMAP TB, except for densely vegetated areas (e.g., tropical forests in low latitudes) with fewer variations in TB and regions affected by RFI (e.g., Eastern Asia, European and Arabian Peninsula). H-polarized TB exhibits a higher correlation with SMAP TB than V-polarized TB, with median R values of 0.913 and 0.903, respectively. The lower correlation coefficients observed in the eastern part of the Sahara compared to the western part can be attributed to the more severe impact of RFI in this region, this phenomenon is consistent with findings from previous studies^42^. The second row of Fig. 6 shows that the V-polarized bias between SMOS and SMAP TB is greater than that of H-polarization. This might be attributed to a systemic bias, which is exacerbated by the RFI issue. Notably, the bias in Eastern Asia and Europe is significantly greater than in other areas. The V-polarized ubRMSD values were reduced significantly compared with RMSD, especially in RFI-affected areas.

### Inter-comparison of SMAP TB with refined TB at 40° and CATDS official TB

**Fig. 7:**
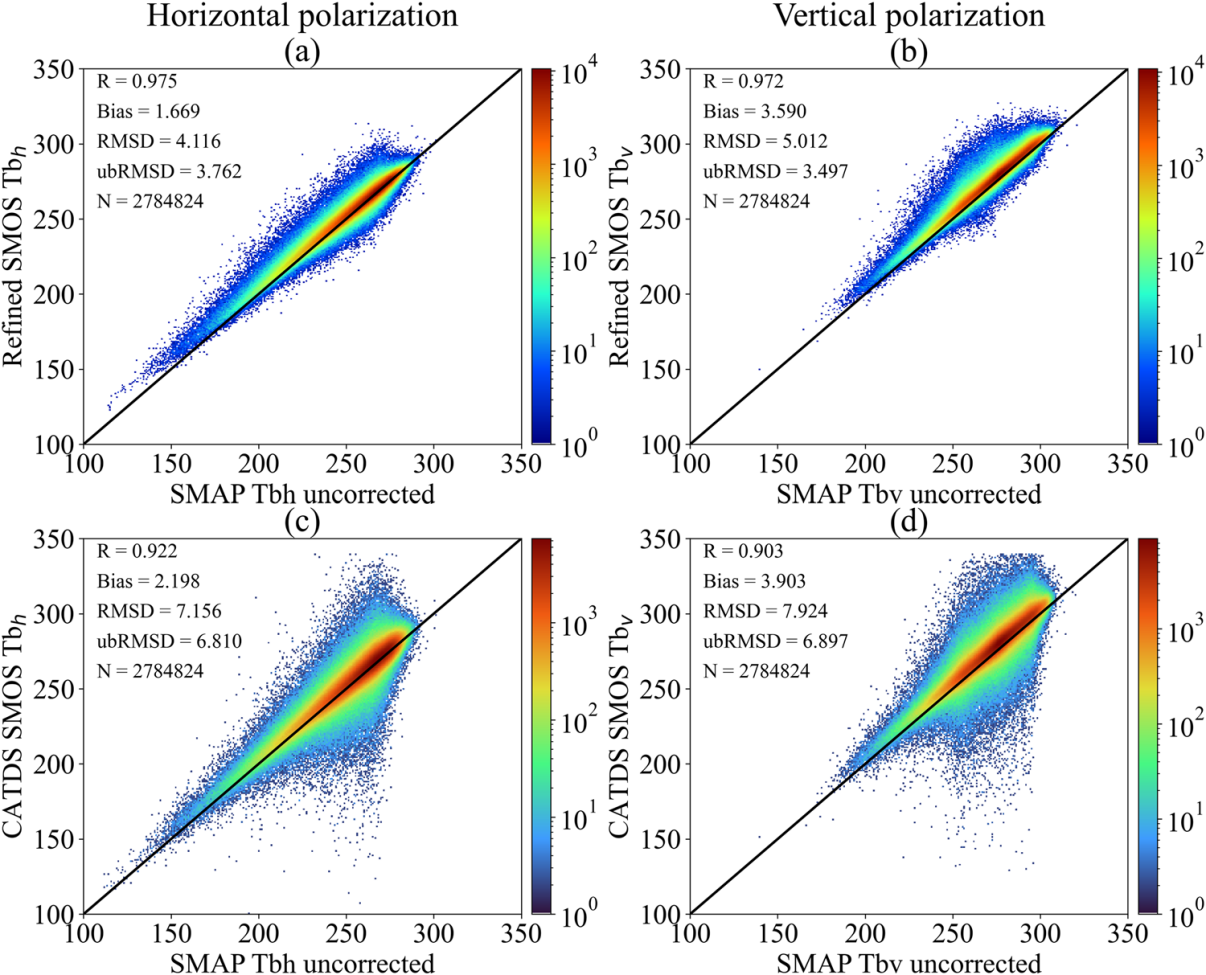
Global land density plot between H-polarization (left column) and V-polarization (right column) of the refined and CATDS OPER SMOS TB at 40° and SPL2SMP TB. (**a,b**) SMAP vs. Refined SMOS; (**c,d**) SMAP vs. CATDS OPER SMOS TB. The data period is from May 25 to December 31, 2021. To be noted, the color-bar is in the log scale.

To further illustrate the RFI-suppressed performance of the refined TB, SMAP TB product was used to compare with the refined and CATDS TB at 40°. Because the SMOS TBs were resampled to EASE-GRID 2.0 36-km, it is impractical to resample the flag in the CATDS TB product, a less strict RFI filtering criterion as described in Eq. (4) was applied before resampling. And the time threshold was set to 3600 seconds in pre-process procedure, it is reasonable to use the SMAP flags (same as Fig. 6) for data filtering. Figure 7 shows the global land spatial-temporal density plot between the two-step refined TB (Fig. 7a,b), the CATDS OPER TB (Fig. 7c,d), and SMAP TB from 25 May to 31 December 2021. It indicates that both the two-step refined TB and the CATDS TB are consistent with SMAP TB in most cases as indicated by the scatter density clustered around the 1:1 line. The SMOS TBs are warmer than SMAP TBs at both polarizations across the globe, which is consistent with previous research^43,44^. For Fig. 7c,d, the SMAP TB is between 250 and 300 K, and the Refined SMOS TB is in good agreement, while the outliers of the CATDS TB below 200 K are mainly attributed to the strong RFI effect (a less strict RFI filtering criterion was used) and the processing methodology, as CATDS L3 TBs were generated without using RFI flags. Though the mean biases are very close (around ~2 K for both refined and CATDS TB at H-polarization, and around ~4 K for both at V-polarization), the two-step regression refined SMOS TB showed a smaller ubRMSD compared to SMAP with a value of 3.762 (3.497) K than CATDS TB with ubRMSD value of 6.81 (6.897) K at H-polarization (and V-polarization). The difference in the ubRMSD performance is primarily attributed to the RFI suppression in the refined SMOS TB, indicating its technical reliability in handling RFI-contaminated data.

### Stability and potential location selection for instrument calibration at L-band

**Fig. 8:**
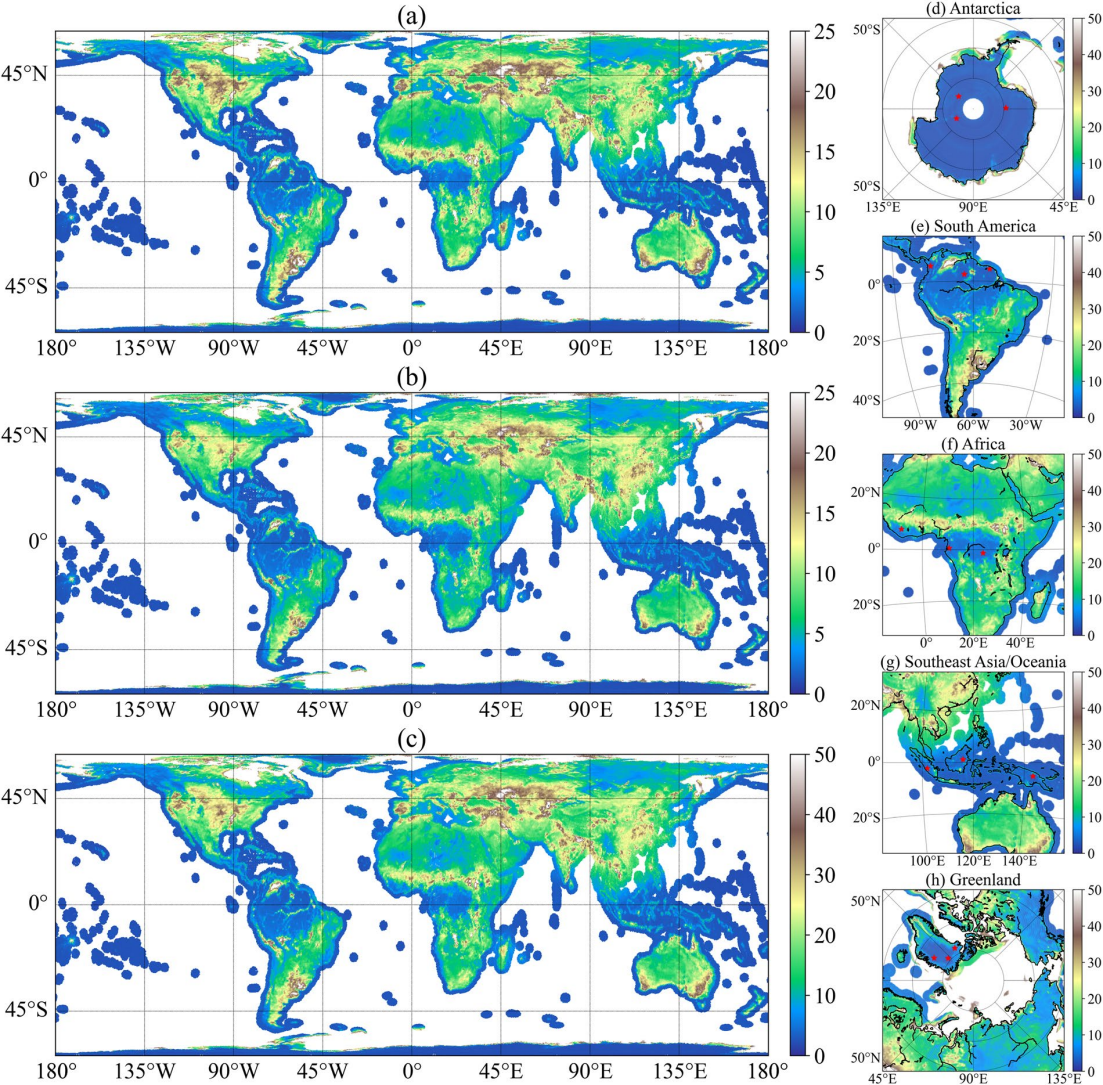
Spatial distribution of standard deviation of refined SMOS TB at 40° in 2021, (**a**) standard deviation of H-polarized TB; (**b**) standard deviation of V-polarized TB, and (**c**) sum of the standard deviation of polarized TB; red stars are selected locations for calibration in (**d**) Antarctica; (**e**) South America; (**f**) Africa; (**g**) Southeast Asia and Oceania and (**h**) Greenland.

To evaluate the stability of SMOS instruments and explore the possibility to have an operative long-term calibration target for subsequent L-band satellites, a simple demonstration was conducted with the refined TB in 2021, and the results are shown in Fig. 8. Figure 8a,b shows the spatial distribution of standard deviation (STD) of the refined SMOS TB at 40° (results for other incidence angles are consistent with those at 40°, and not shown in the study). Small STDs of TBs are predominantly observed in high-latitude areas, such as Greenland and Antarctica, as well as densely vegetated regions like the Amazon Forest and tropical forests in Malaysia at lower latitudes. These regions exhibit high stability in TB, implying that the SMOS instruments are functioning well. Consequently, these areas have the potential to serve as reliable calibration targets at the L-band due to their minimal TB variations. However, it is essential to note that that even in these stable areas, TB variations can still occur due to temporal changes in soil moisture and vegetation optical depth, especially in dense forests. This variation should be considered when using forests as calibration targets. Figure 8d–h focuses on selected DGGs with the minimum sum of standard deviation on continents considering factors such as distance to the coastline, and land cover homogeneity. Detailed information about these selected calibration DGGs is listed in Table 4. These locations are identified as potential areas for instrument calibration. For example, DGG 1152189 in Fig. 8(e) is situated near one of the zones in the Amazon Forest that have been used for SMOS calibration^10,45^. The stability of TBs is particularly high all over Antarctica (Fig. 8d). The Dome-C was selected as the campaign for calibration in previous studies^10,45,46^. The mean values of Dome-C’s H- and V-polarized TBs were 187.059 K and 211.509 K, respectively, with standard deviations of 1.033 K and 1.143 K. In the current study, the selected calibration DGGs are not located in Dome-C. As shown in Table 4, these selected DGGs exhibit even smaller standard deviations in TB compared to Dome-C. Additionally, the mean TB of the selected DGGs is higher than that observed at the Dome-C. These selected reference targets may serve as “warm” and “cold” targets for the calibration of the future Terrestrial Water Resources Satellite (TWRS) over the land area^38^.

**Table 4:** The selected DGG information for calibration in Fig. 8, the mean and STD of TB are all calculated using the data at 40° in 2021.

DGG ID	Longitude	Latitude	Mean of TBh	STD of TBh	Mean of TBv	STD of TBv	Fig. 8
6249545	−139.533	−83.642	189.722	0.893	221.863	0.919	(d)
6252745	−1.713	−79.373	201.231	0.791	222.165	1.032	(d)
7158754	150.772	−83.700	194.764	0.744	221.250	0.956	(d)
1112149	−76.255	5.986	271.502	1.190	275.522	1.201	(e)
1152189	−63.473	2.700	276.502	1.398	280.326	1.355	(e)
1166606	−53.745	4.752	276.248	1.408	280.799	1.235	(e)
2037509	−8.083	7.631	271.963	2.029	279.377	2.123	(f)
2126717	10.423	0.619	274.977	1.806	279.303	1.432	(f)
2185183	23.861	−1.378	278.022	1.856	282.437	1.801	(f)
8002802	101.870	−2.224	272.918	1.279	277.274	1.149	(g)
8061254	115.869	1.225	275.716	1.259	279.840	1.579	(g)
8183696	143.350	−5.151	271.729	1.152	274.839	1.027	(g)
48463	−60.257	77.767	170.519	1.823	207.350	1.807	(h)
36185	−41.228	79.036	191.604	1.126	222.506	1.046	(h)
39808	−29.860	75.202	195.219	1.435	224.356	1.191	(h)

TBh indicates TB at H-polarization, and TBv indicates TB at V-polarization.

## Usage Notes

In this study, we have presented an RFI-suppressed SMOS multi-angular TB dataset^32^ spanning over a decade. The two-step regression method proposed by Zhao *et al*.^20^ was used to refine or interpolate the multi-angular SMOS TB affected by field aliasing and RFI. The resulting refined TBs in DGGs were then resampled to the EASE-GRID 2.0 projection, with two grid sizes of 25 km and 36 km, using a Hamming window approach with a footprint of 43 km.

As described in the previous section, this refined SMOS TB dataset^32^ has demonstrated great RFI-suppression performance. It exhibits strong agreement with SMAP TB at 40°, and the corresponding emissivity derived from the refined TBs shows a better relationship with *in-situ* soil moisture compared to the CATDS TB in most cases. This dataset^32^ can be used to retrieve essential land parameters such as soil moisture and snow density etc. Moreover, it is expected to provide opportunities for advancing scientific applications over land.

## Data Availability

The software and codes for processing the collected data and for plotting the figures are conducted in Python 3.7 and included in the dataset at 10.11888/Terre.tpdc.300406 or https://cstr.cn/18406.11.Terre.tpdc.300406. And they are also available on GitHub: https://github.com/thimpeng/RFI-Suppressed_SMOS_L-band_multi-angular_TB_Refinement.
